# Impact of ChatGPT usage on nursing students education: A cross-sectional study

**DOI:** 10.1016/j.heliyon.2024.e41559

**Published:** 2024-12-31

**Authors:** Alberto Gonzalez-Garcia, David Bermejo-Martinez, Ana Isabel Lopez-Alonso, Bibiana Trevisson-Redondo, Cristian Martín-Vázquez, Silvia Perez-Gonzalez

**Affiliations:** a CUHUSAL-BPS Teaching Innovation Group, Faculty of Health Sciences, Nursing and physiotherapy department, Universidad de León, 24007, León, Spain; b CUHUSAL-BPS Teaching Innovation Group, Faculty of Health Sciences, Nursing and physiotherapy department, Campus de Ponferrada, Universidad de León, 24401, León, Spain; c Faculty of Health Sciences, Nursing and physiotherapy department, Universidad de León, 24007, León, Spain

**Keywords:** ChatGPT, Nursing education, Artificial intelligence, Academic performance, Technological innovations, Student perceptions, Nurse

## Abstract

**Background:**

The use of artificial intelligence tools, such as ChatGPT, is on the rise in nursing education. In the field of healthcare, ChatGPT can offer unique opportunities to enhance the learning and clinical practice of nursing students. However, it is still necessary to explore how this tool affects students' performance and perception in their nursing education.

**Objective:**

The objective of this study was to evaluate the impact of ChatGPT on nursing students' education and determine how it influences their learning outcomes.

**Design:**

This study employed a quantitative cross-sectional design.

**Setting:**

The study was conducted in the Bachelor of Nursing program at the University of León, Spain.

**Participants:**

Ninety-eight nursing students enrolled in the Nursing Care and Services Management course during the second semester of 2024 participated in the study.

**Methods:**

Data were collected using three validated questionnaires that assessed sociodemographic characteristics, knowledge of artificial intelligence, and perceptions of using ChatGPT as an educational tool. The data were analyzed using IBM SPSS Statistics, version 29.1.

**Results:**

Students who used ChatGPT showed a significant improvement in their academic grades (p < 0.05). Additionally, 89.5 % of the students reported significant improvements in their academic performance. Women perceived ChatGPT as especially useful for completing academic tasks (85.14 % versus 50.00 % in men, p = 0.003). A positive correlation was observed between prior use of ChatGPT and GPA (ρ = 0.240, p = 0.026).

**Conclusions:**

ChatGPT is a valuable tool that enhances the learning and satisfaction of nursing students. Its integration into nursing education programs not only boosts academic performance but also promotes the adoption of technological innovations in professional training. Continuous incorporation of AI tools in education is recommended to improve academic outcomes and prepare students for evolving healthcare environments.

## Highlights

• Nursing students showed significant improvements in academic performance using ChatGPT.
• 89.5 % of nursing students reported that ChatGPT significantly enhanced their academic performance.
• Women nursing students found ChatGPT particularly useful for completing academic tasks.
• There is a positive correlation between prior use of ChatGPT and higher GPA among nursing students.
• Continuous integration of AI tools like ChatGPT is recommended to enhance nursing education.

## 1 Introduction

**Fig. 1:**
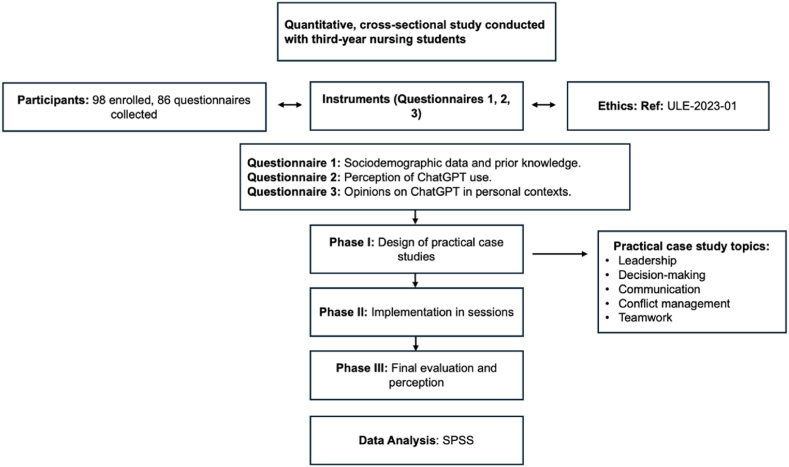
Overview of the methodological design.

Change and improvement are the concepts that should guide innovation in education and learning [[1], [2], [3]]. Thus, we can define innovation in higher education as the implementation of an idea that generates a planned change in the educational process, which, in turn, improves learning objectives [4,5]. Currently, Generative Artificial Intelligence (GAI) has changed various facets of our lives, such as access to information, communication, and interaction between people [6]. Consequently, younger generations have made GAI a staple in their daily routines, including its use for studying and knowledge acquisition [7]. Therefore, we should consider these tools as key to educational innovation, as they act as facilitators in the development of knowledge ([3,8]; J. [9]) (see Fig. 1).

In this sense, university education has undergone significant changes due to the transformative impact of technological advances in this century [10,11]. Among these changes, generative AI (ChatGPT) has established itself as a virtual assistant in the university setting, offering support and guidance to students during their academic process [12]. Text-generated language models, such as ChatGPT, can offer simulated situations to students to advance the development of competencies such as decision-making, communication, or conflict management [[13], [14], [15], [16], [17]]. In addition, its use increases student engagement and interaction, making it a valuable learning methodology [18,19]. Complementarily, technologies such as Virtual Reality (VR) have demonstrated significant potential to enhance educational experiences by providing immersive and interactive learning environments [20]. These tools not only foster engagement and motivation but also enable the creation of simulated virtual environments that offer safety, remote accessibility, and cost reduction [21].

ChatGPT has revolutionized the lives of students, making it essential to evaluate the changes this platform brings to their academic performance [22]. For this reason, we must distinguish several aspects that link ChatGPT and education ([23,24]; L. [25]): (1) the practical uses students make of it for their training; (2) the understanding of its capacity and application in teaching methodologies; and (3) the awareness of teachers and students regarding the impact of ChatGPT on university training.

On the other hand, among faculty members, as the use of generative artificial intelligence (GAI) learning tools such as ChatGPT becomes increasingly important in university teaching, debates persist regarding their impact on learning activities and academic outcomes [[26], [27], [28]]. Some research, such as that conducted by McNeil [29], indicates that only half of the faculty perceive the integration of technology as valuable. However, other studies highlight the benefits of using ChatGPT for university faculty [7,13,22], including facilitating learning processes, increasing faculty productivity, and improving the overall functioning of the university [[30], [31], [32]]. In this context, the integration of activity-based learning (ABL) with ChatGPT has demonstrated significant potential for enhancing student engagement and academic outcomes. ABL promotes critical thinking and problem-solving skills, while ChatGPT serves as a valuable assistant in the learning process, streamlining tasks and providing timely support [33].

Therefore, our research focuses on the introduction of innovative didactic methods in nursing education, utilizing GAI applications such as ChatGPT to analyze how its use impacts the learning process and educational outcomes. ChatGPT is presented as an innovative tool that can facilitate the development of competencies required by nurses today [[34], [35], [36], [37], [38]].

These technologies, such as ChatGPT, are emerging as a new field of research, particularly regarding their impact on academic performance. However, despite the growing interest in generative artificial intelligence applications in university education, there is limited evidence of their impact on nursing students' education. In particular, the relationship between the perceived usefulness of ChatGPT and its influence on student achievement and critical thinking skills remains poorly understood. This gap is especially relevant in educational contexts that require a balance between advanced technologies and healthcare skills training. Therefore, it is essential to deepen the understanding of how tools such as ChatGPT can be ethically and effectively integrated into the nursing curriculum, fostering not only academic improvement but also the development of competencies tailored to healthcare settings.

For all these reasons, we asked ourselves: What is the impact of ChatGPT use in nursing education? To address this question, we set out to evaluate the impact of ChatGPT on the education of nursing students. Specifically, the study aims to determine how the incorporation of ChatGPT influences the educational process and the learning outcomes of nursing students.

The remainder of the article is divided into four main sections: Section 2 provides a brief literature review summarizing the theoretical foundations of the research. Section 3 describes the methodology of the research. The analysis of the results is presented in Section 4. Section 5 discusses the results, implications, limitations, and suggestions for future research. Finally, Section 6 presents the conclusions of the research.

## 2 Literature review

The current state of ChatGPT usage in the process of university training reflects a growing desire for its inclusion, driven by the increasing digitization and evolution of the academic environment [18,39,40].

As for the effect of ChatGPT on different aspects of university education, the literature review highlights how ChatGPT has revolutionized the educational field by introducing a dynamic and personalized approach that encourages interactivity and adaptation in learning [41,42]. Its ability to detect errors, synthesize information, and streamline complex academic processes positions it as a key tool in transforming workflows and improving academic outcomes, contributing to a more efficient and enriching environment [43,44]. Another important aspect that emerges from the literature review is the capability of AI to predict academic performance, as well as its potential to transform teaching methodologies and the development of educational materials [[45], [46], [47]].

Regarding the improvement of the educational process, different research studies have highlighted the transformative potential of AI and tools such as ChatGPT to personalize learning, automate processes, and improve educational outcomes, as well as the importance of creating collaborative environments and ensuring ethical training in the use of these technologies [48,49]. In this sense, Shahzad et al. [50] pointed out how the integration of generative AI and social networks has a positive impact on academic performance and psychological well-being of university students. Similarly, Gao et al. [51] indicated that generative AI improves motivation, learning desire, and future beliefs, although its effect on self-efficacy is limited, highlighting the importance of balancing technology with educational methods. In addition to its impact on academic performance, artificial intelligence and machine learning algorithms, such as Random Forest and decision trees, have proven effective in predicting student enrollment and developing school classification models [52].

In terms of academic performance, ChatGPT, as an AI tool, improves academic outcomes by providing personalized, rapid, and interactive feedback, fostering student engagement and independent learning [43,53]. Its constant availability and ability to adapt to individual learning paces promote a greater understanding of content and better academic results [54,55]. Meanwhile, Amjad et al. [56] noted that mobile learning, together with ChatGPT and social networks, enhances academic performance by facilitating accessibility, personalization, and interactive participation in learning. Similarly, Komba [57] analyzed the impact of ChatGPT, highlighting significant improvements in study habits, academic performance, and comprehension of learning materials. Additionally, Almulla et al. [58] evaluated the impact of ChatGPT, identifying significant enhancements in academic performance and research skills.

In relation to the personalization of learning, ChatGPT can provide tailored responses to the specific needs of students, thereby improving their academic performance [26,28,43]. In addition, it assists students in focusing their efforts through its ability to perform assessments and provide feedback on their progress [[59], [60], [61]]. However, its usefulness depends on proper integration into teaching plans, which requires additional research to maximize its impact [27,62].

Regarding the perception and adoption of ChatGPT, various studies have emphasized the importance of factors such as perceived usefulness, technological features, and individual characteristics in shaping the attitudes of students toward tools like ChatGPT, stressing the importance of aligning technology with educational needs [63,64]. Moreover, the cognitive benefits it offers, such as adaptive learning and intelligent tutoring, reinforce its role in skill development and knowledge acquisition [[65], [66], [67]]. However, these studies also highlight the need for digital literacy and continuous updates to ensure the effective and ethical use of the tool [[45], [46], [47]].

For better understanding, the following is a list of the main abbreviations used in this study:

**AI:** Artificial Intelligence.

**ABL:** Activity-Based Learning.

**BSN:** Bachelor of Science in Nursing.

**GPA:** Grade Point Average.

**GAI:** Generative Artificial Intelligence.

**SPSS:** Statistical Package for the Social Sciences.

**VR:** Virtual Reality.

## 3 Methods

### 3.1 Study design

This study employed a quantitative cross-sectional design to examine the influence of ChatGPT on the education of nursing students and their perception of its usefulness in training.

### 3.2 Study settings

This study was conducted in the Bachelor of Science in Nursing (BSN) program at the University of León, Spain.

### 3.3 Participants

The target population included 98 nursing students enrolled in the Nursing Care and Services Management course during the second semester of 2024. The sample size was determined based on the expected variability. Considering a variability rate of 50 % and a total of 98 students enrolled in the course, a minimum sample size of 79 participants was calculated, allowing for the estimation of proportions with a 5 % margin of error and a 95 % confidence level. Finally, 86 questionnaires were collected, exceeding the required minimum sample size.

### 3.4 Research instruments

The data for this research were collected using three newly developed questionnaires, which were internally validated by a group of experts.

Questionnaire (1) This questionnaire was created by the researchers based on a literature review. It included:

(a) Sociodemographic data of the students, including age, gender, place of residence, employment status, academic performance (GPA), and hours dedicated to studying per week.
(b) Knowledge of artificial intelligence among students. This section explores the prior use of tools similar to ChatGPT and the familiarity with other content-generating AI tools, using multiple-choice questions. Response options include specific uses of ChatGPT ("Support with academic tasks," "Content generation," etc.) and familiarity with AI tools ("I have used it," "I have not used it," "I am not familiar with these tools").

The validation process involved five professors from the Department of Nursing and Physiotherapy, along with final-year nursing students not enrolled in the course. Initially, the objectives and content of the research, as well as the purpose of their task, were explained to them. They were asked to identify potential issues such as errors in the questions, difficulty understanding the content, ambiguity, or any doubts that participants might encounter while responding. Additionally, they were asked to evaluate the duration of the questionnaire, considering fatigue or stress during completion.

As a result of this validation process, adjustments were made to the wording and formulation of certain questions, as well as to the overall structure of the questionnaire. These refinements ensured that each question was clear, relevant, and capable of effectively capturing the intended insights. The Cronbach's alpha coefficient indicated a reliability of 0.87.

Questionnaire (2) Perception of the use of ChatGPT as a training tool. This questionnaire was developed by the researchers to evaluate students' perceptions and experiences with ChatGPT in their academic training. It consisted of 10 questions on a 1–5 Likert scale, where 1 = not at all satisfied and 5 = completely satisfied, and 12 questions with dichotomous (Yes/No) or multiple-choice formats. The questionnaire explored the usefulness of ChatGPT for understanding management concepts, improving academic performance, and its potential application in professional nursing practice. It also assessed overall satisfaction with ChatGPT as an educational tool.

The validation of this questionnaire was carried out with the same group of experts as in Questionnaire (1), using identical criteria. These experts evaluated the clarity, relevance, and comprehensibility of the questions, ensuring consistency in the validation process. The Cronbach's alpha coefficient was found to be 0.79.

Questionnaire (3) Students' opinion on the use of ChatGPT. This questionnaire was developed by the researchers and consisted of 7 multiple-choice questions, with response options such as "Yes," "No," and "I am not sure," as well as one open-ended question for additional comments and suggestions. It aimed to explore students' perspectives on ChatGPT's use in their personal and family environments, including familiarity, interest, and practical applications.

The validation of this questionnaire was conducted by the same group of experts as in the previous questionnaires, following identical criteria to ensure clarity, relevance, and comprehensibility. The process ensured the quality of the instrument, with adjustments made as necessary to refine the questions. The questionnaire demonstrated reliability, with a Cronbach's alpha coefficient of 0.89.

### 3.5 Ethical Considerations

Ethical approval for this study was granted by the Research Ethics Committee of the University of León (Ref: ULE-2023-01). All participants were informed about the purpose of the study and assured that their participation was entirely voluntary, with information provided about their right to withdraw. Informed consent was obtained at the beginning of the online questionnaire, using the informed consent model approved by the University of León, which explained that the submission of responses implied consent to use their responses in the study. The collected data were anonymized and securely stored on a protected online platform.

The results are reported in aggregate form, ensuring that individual participants cannot be identified. All data were securely stored on password-protected laptops, with exclusive access for the research team members.

### 3.6 Plan and data Collection

The planning, design, and implementation of the program lasted eight months, from November 2023 to June 2024. Students enrolled in the course were invited to complete a Google form, which was set up as the platform for conducting the questionnaires. The link to the questionnaire was provided via institutional email, and access was facilitated through a QR code made available on the official Moodle platform of the University of León.

This study was conducted in three consecutive phases.

**Phase I**: Eight case studies were designed related to the essential competencies of the course, such as communication, leadership, conflict management, decision-making, ethical principles, teamwork, change management, and innovation. Each case study provided a detailed explanation of the dynamics to follow for resolving the case. Subsequently, the case study presented the context, challenges, and characters of the case, along with their different functional roles. The case illustrated everyday situations that a nurse or a nurse manager might face during their professional practice. Additionally, structured guidance was provided on how to use ChatGPT to resolve the case.

**Phase II:** During the practical sessions of the course, the case and dynamics were explained to the students. They were also provided with guidelines for working with ChatGPT. The time allocated to resolve the case study was 120 min, after which the students had to present their resolution along with the use of ChatGPT to reach that resolution.

**Phase III:** After the completion of the practical sessions and at the end of the course, the resolution of the case studies using ChatGPT was evaluated. In June, the students completed a questionnaire regarding their perception of the usefulness of ChatGPT for improving their knowledge and skills related to the Nursing Care and Services Management course.

### 3.7 Data analysis

The data collected through the online questionnaire were analyzed using IBM SPSS Statistics software, version 29.1 for Mac. The data analysis primarily included closed-ended questions. Descriptive statistics were used to summarize the sociodemographic characteristics of the participants and their responses to the questionnaire items, covering frequencies, means, and standard deviations.

To examine the differences in perceptions and the use of ChatGPT according to various sociodemographic characteristics, several additional analyses were conducted. Analysis of Variance (ANOVA) was used to compare differences between groups in terms of categorical variables such as gender, employment status, and place of residence. Additionally, Student's t-tests were applied to compare means between two different groups, such as average academic performance by gender.

A correlation analysis was conducted to examine the relationships between variables using Pearson's r correlation coefficient and Spearman's rho. For ordinal variables, Fisher's exact test was utilized.

Finally, Chi-square analyses were employed to evaluate associations between categorical variables, such as the perceived usefulness of ChatGPT and the demographic characteristics of the participants. All statistical analyses were performed with a significance level set at p ≤ 0.05.

## 4 Results

### 4.1 Participants sociodemographic characteristics

A total of 86 third-year nursing students participated in this study. The average age of the participants was 23.01 years (SD = 6.983), ranging from 20 to 52 years. The majority of participants were women (86.05 %), which accurately reflects the current demographic characteristics of nursing students [68]. Additionally, 5.8 % of the participants reported having prior studies. No statistically significant differences in academic performance were observed concerning gender, employment status, place of residence, or academic average (Table 1).

**Table 1:** Sociodemographic data.

Characteristic	Value	n	%	Age Mean ± SD	Grade Point Average (GPA) Mean ± SD	t/F (GPA)	P (GPA)
**Age**				23.01 ± 6.983			
**Gender**	Female	74	86.05	23.12 ± 7.13	7.8 ± 0.9	−1.959	0.053
	Male	12	13.95	22.33 ± 6.223	7.7 ± 0.9		
**Employment Status**	Full-time student	70	81.40	22.84 ± 7.04	7.8 ± 0.8	0.742	0.479
	Student and part-time worker	6	6.98	22.83 ± 5.98	7.7 ± 0.9		
	Student and full-time worker	10	11.63	24.30 ± 7.63	8.0 ± 1.1		
**Place of Residence**	Urban area	71	82.56	23.01 ± 7.14	7.7 ± 0.9	1.64	0.205
	Suburban area	5	5.81	20.40 ± 0.55	7.5 ± 0.5		
	Rural area	10	11.63	24.30 ± 7.63	7.9 ± 0.9		
**Prior Studies**	Yes	5	5.8				
	No	81	94.2				
**Grade Point Average (GPA)**	5–6	2	2.33	35 ± 21.21			
	6–7	10	11.63	21.70 ± 2.63			
	7–8	51	59.30	22.27 ± 5.76			
	8–9	22	25.58	24.32 ± 8.72			
	9–10	1	1.16	21 ± 0			

### 4.2 Previous use of ChatGPT

Regarding the frequency of ChatGPT and other content generation tools used by students before the course, Table 2 presents the various applications and their corresponding usage frequencies as percentages.

**Table 2:** Frequency of ChatGPT and content generation tools usage before the course.

Functionality	Frequency of Use (%)
**Prior Chat Use**	59.3 %
**Academic Use**	80.4 %
**Content Generation Use**	62.7 %
**General Information Use**	52.9 %
**Problem-Solving Use**	21.6 %
**Image Generators**	24.4 %
**Video Generators**	9.3 %
**Music Generators**	3.5 %

The results indicate that the academic use of ChatGPT is the most frequent, with 80.4 % of students reporting its use for educational purposes. Other common uses include content generation (62.7 %) and obtaining general information (52.9 %).

Subsequently, it was analyzed whether these variables were related to academic performance. Table 3 shows the statistically significant correlations found.

**Table 3:** Statistically significant correlations with academic record.

Variables	Correlation Coefficient (ρ)	Significance (2-tailed)
**Prior ChatGPT Use ∗ GPA**	0.240∗	0.026
**GPA ∗ Image Generation**	−0.249∗	0.029
**Academic Assistance ∗ General Information**	0.425∗∗	0.002
**Image Generation ∗ Academic Assistance**	−0.461∗	0.035
**Video Generators ∗ Image Generation**	0.470∗∗	0.000
**Music Generators ∗ Video Generators**	0.594∗∗	0.000
**Music Generators ∗ Image Generation**	0.334∗∗	0.002

**Table 4:** Perceived usefulness of ChatGPT for academic concepts.

Variable	Gender	Very Useful (%)	Useful (%)	Neutral (%)	Slightly Useful (%)	Not Useful (%)	p-value (Fisher)
**Useful for understanding course concepts**	Female	54.05	35.14	9.46	1.35	0	0.508
	Male	66.67	33.33	0	0	0	
**Content comprehension**	Female	54.05	45.95	0	0	0	0.79
	Male	58.33	41.67	0	0	0	
**Understanding practical cases**	Female	93.24	6.76	0	0	0	0.89
	Male	91.67	8.33	0	0	0	
**Exam preparation**	Female	28.38	71.62	0	0	0	0.73
	Male	33.33	66.67	0	0	0	
**Completing assignments**	Female	85.14	14.86	0	0	0	0.003
	Male	50.00	50.00	0	0	0

**Table 5:** Correlation between GPA and perceived usefulness.

Variable	Perception_Utility
**Grade Point Average**	0.450
**Perception_Utility**	1.000
**Sig. (2-tailed)**	0.001

The findings suggest a weak positive correlation between prior use of ChatGPT and Grade Point Average (GPA) (ρ = 0.240, p = 0.026). While the relationship is statistically significant, the low strength of the correlation indicates that other factors likely contribute to GPA. Additionally, a positive correlation was found between academic assistance and obtaining general information (ρ = 0.425, p = 0.002).

Image generation shows a negative correlation with GPA (ρ = −0.249, p = 0.029) and with academic assistance (ρ = −0.461, p = 0.035), which may indicate that students with better GPAs tend to focus less on creative activities not directly related to their studies. Video and music generators also show significant correlations with image generation, suggesting that these tools are used complementarily.

### 4.4 Perceived improvement in academic performance

Regarding the perceptions of students on the improvement in their academic performance in the management course through the use of ChatGPT, 89.5 % of participants reported significant or very significant improvements. Conversely, 1.2 % indicated that the use of ChatGPT hindered their learning process (Table 6).

**Table 6:** Perceived improvement in academic performance.

Response	Frequency	Percentage (%)	Cumulative Percentage (%)
**Yes, significantly**	40	46.5	46.5
**Yes, to some extent**	37	43.0	89.5
**No, no changes**	7	8.1	97.7
**No, it hindered my performance**	1	1.2	98.8
**I am not sure**	1	1.2	100.0

Next, the relationship between the perception of academic performance improvement and the perceived usefulness of ChatGPT in the course was analyzed. The statistically significant results are shown in Table 7.

**Table 7:** Perception of academic performance improvement and perceived usefulness.

Cross Variable	Pearson Chi-Square	Degrees of Freedom (df)	Significance (p-value)	Number of Valid Cases
**Academic Performance Improvement ∗ Useful for understanding concepts**	2.688	2	0.002	86
**Academic Performance Improvement ∗ Relating management concepts (course)**	4.927	2	0.001	86
**Academic Performance Improvement ∗ Relating management concepts (practice)**	4.927	2	0.001	86

These results suggest that students who perceive ChatGPT as useful for understanding course concepts and for relating management concepts, both in theory and practice, also perceive a significant improvement in their academic performance. This finding highlights the importance of the perceived usefulness of technological tools like ChatGPT in the learning process and the perceived improvement in academic performance by students.

### 4.3 Perception of usefulness of ChatGPT

Regarding the usefulness of ChatGPT, Table 4 shows the degree of usefulness for various academic concepts related to the management course. Additionally, Fisher's exact test was conducted for each of the analyzed areas of usefulness.

The results show that students consider ChatGPT to be a very useful tool for the course. No significant differences were found in the perceived usefulness of ChatGPT in most of the analyzed areas, such as understanding course concepts, content comprehension, understanding practical cases, and exam preparation.

However, a significant difference was found in the perceived usefulness of ChatGPT for completing academic assignments (p = 0.003). In this category, a higher percentage of women perceive ChatGPT as very useful compared to men (85.14 % vs. 50.00 %). This finding suggests that ChatGPT might be especially useful for women in completing academic assignments.

It was also analyzed whether the students' GPA was correlated with their perception of usefulness (Table 5).

The correlation between GPA and the perceived usefulness of ChatGPT is ρ = 0.450 with a p-value of 0.001. This indicates that there is a significant correlation between GPA and the perceived usefulness of ChatGPT (p < 0.05). This suggests that as GPA increases, so does the perception of ChatGPT's usefulness among nursing students.

## 5 Discussion

The objective of this study was to evaluate the impact of using ChatGPT in the training of nursing students. Utilizing a cross-sectional quantitative design, the research was conducted among third-year students in the Bachelor of Nursing program at the University of León, Spain. The results suggest that the incorporation of ChatGPT in the educational process is not only perceived as a useful tool by students but may also be associated with improvements in academic performance, particularly in completing academic assignments. This demonstrates the need for changes in classroom training that promote and support critical reasoning through the integration of innovative teaching strategies, such as those provided by AI [69].

The findings indicate that the academic use of ChatGPT is the most frequent, at 80.4 %. The high frequency of academic use of ChatGPT suggests that students find this tool to be a reliable and efficient resource for their studies [70]. Currently, there is a growing integration of ChatGPT in academic training, as students find this tool to be a versatile resource that facilitates not only learning but also the creation and access to relevant information [30,71,72]. In this regard, Mamo et al. [73] highlighted how the integration of advanced technologies such as AI significantly enhances the learning experience. Furthermore, the high valuation of AI usage aligns with the growing recognition of this technology as an agent of change in healthcare, underscoring the importance of integrating AI training into nursing degree curricula [[74], [75], [76]].

The results of this study indicated a statistically significant positive correlation between prior use of ChatGPT and average GPA (p < 0.05), as well as between its use for academic tasks and obtaining general information (p < 0.01). This outcome aligns with previous studies that highlight the effectiveness of educational interventions using AI to enhance knowledge and attitudes of future professionals towards new technologies [73,74].

The findings of this study reveal a significant difference in the perceived usefulness of ChatGPT for completing academic assignments, with a higher percentage of women (85.14 %) considering it very useful compared to men (50.00 %) (p = 0.003). Additionally, a significant and moderate correlation was observed between GPA and the perceived usefulness of ChatGPT and its use during the course (ρ = 0.450, p = 0.001). While this suggests that students with higher academic performance are more likely to perceive ChatGPT as useful, it is important to note that other correlations found in the study, such as the one between prior ChatGPT use and GPA (ρ = 0.240,p = 0.026), were statistically significant but weak, highlighting the influence of other factors on academic performance. Various studies indicate that students who consider ChatGPT a useful tool experience a greater sense of control and competence in their learning, which is reflected in a better perception of their academic performance [70,[77], [78], [79]]. Furthermore, students who use ChatGPT feel a greater ability to manage their academic tasks, particularly in completing assignments [80]. The significant difference in perceived usefulness between women and men suggests that ChatGPT may be particularly empowering female students, helping them overcome barriers and develop confidence in their academic abilities [81]. This result is consistent with previous studies that have demonstrated that educational technologies can have a positive impact in helping to reduce gender gaps in academic performance [82,83].

Additionally, the significant correlation between GPA and the perceived usefulness of ChatGPT indicates that students with higher academic performance are more likely to recognize and utilize the capabilities of ChatGPT to further enhance their learning [84]. This reinforces the notion of technological empowerment, where students who already have a high academic performance benefit more from technological tools, consolidating their position and enhancing their academic achievement [9,81,85]. The ability of ChatGPT to provide quick and accurate answers to academic questions enables students to approach their studies with greater confidence and efficiency [86]. This immediate access to information reduces dependence on traditional resources and promotes greater autonomy in learning, which is crucial for developing critical skills in the field of nursing [86].

The research results also highlighted that 89.5 % of students perceive a significant or very significant improvement in their academic performance in the management course through the use of ChatGPT. The relationship between the perceived usefulness of ChatGPT and the perception of a substantial improvement in academic performance in the course (p < 0.05) is noteworthy. In this regard, Labrague et al. [87] revealed that the adoption of artificial intelligence tools in education significantly improves the knowledge and skills of students, facilitating the understanding and application of theoretical and practical concepts in real-world settings. In line with this, our results suggest that the integration of ChatGPT not only fosters a deeper understanding of concepts but also strengthens the ability of students to relate and apply management course concepts in practical situations. This translates into the perception of improved performance in the course. By providing students with these resources, they are able to better understand the concepts and apply them in practice [71,88]. This is essential for the development of competencies in higher education, as students feel they have the necessary tools to overcome academic challenges and improve their performance [89,90].

### 5.1 Limitations

Firstly, the cross-sectional design of the study limits the ability to establish causal relationships between the identified factors. Additionally, the study relied on self-reported data, which may be subject to response biases. Moreover, the study was conducted with a specific sample of nursing students in a particular course, which may limit the generalizability and applicability of the results to other populations of nursing students. It would be beneficial to replicate this study in other nursing courses and at other universities. Furthermore, while the study found a statistically significant correlation between prior ChatGPT use and GPA, the strength of this correlation was weak (ρ = 0.240), suggesting that other factors likely influence academic performance. Future studies should explore these additional factors and investigate the mechanisms through which ChatGPT and similar tools might contribute to academic outcomes.

### 5.2 Future research

Future research should continue to investigate how the integration of ChatGPT and other AI technologies in the nursing educational environment influences the development of specific competencies and the professional preparation of students. Future studies could focus on the long-term impact of these tools on clinical practice and the adaptation of students to constantly changing healthcare environments. Additionally, it would be beneficial to explore the effectiveness of ChatGPT in different disciplines and educational contexts to determine its applicability and advantages in a broader range of academic and professional scenarios.

## 6 Conclusions

This research evaluated the impact of ChatGPT on nursing training and reached several important conclusions.

First, the results indicate that ChatGPT is perceived as a useful and beneficial tool that significantly improves students' academic performance and understanding, enriching the learning experience.

Second, a significant group of students reported substantial improvements in their academic performance by using ChatGPT.

Third, the analysis showed that women perceive greater benefits from ChatGPT compared to men, which could be related to greater confidence in adopting educational innovations.

Fourth, it was observed that students with better academic records take more effective advantage of ChatGPT for their learning.

Finally, these findings highlight the need to integrate tools such as ChatGPT into educational programs.

To optimize the impact of these tools, educational institutions should consider policies that ensure equitable access, training in their use, and the development of complementary resources to support both student learning and well-being.

### 6.1 Implications for nurse education

The integration of ChatGPT in nursing education enhances learning and prepares future professionals for evolving AI technologies in healthcare. It fosters a culture of innovation essential in a rapidly changing sector. Ongoing research is crucial to understand its impact on education and professional practice.

## CRediT authorship contribution statement

**Alberto Gonzalez-Garcia:** Writing – review & editing, Writing – original draft, Software, Resources, Project administration, Methodology, Investigation, Formal analysis, Data curation, Conceptualization. **David Bermejo-Martinez:** Writing – review & editing, Supervision, Software, Conceptualization. **Ana Isabel Lopez-Alonso:** Writing – review & editing, Validation, Conceptualization. **Bibiana Trevisson-Redondo:** Writing – review & editing, Validation, Methodology, Conceptualization. **Cristian Martín-Vázquez:** Writing – review & editing, Methodology, Investigation, Conceptualization. **Silvia Perez-Gonzalez:** Writing – review & editing, Methodology, Investigation, Conceptualization.

## Declaration of generative AI and AI-assisted technologies in the writing process

“During the preparation of this work, the author(s) used ChatGPT to improve the grammar and spelling in English. After using this tool/service, the author(s) reviewed and edited the content as needed and take(s) full responsibility for the content of the publication.”

## Funding

Funding was received for this manuscript by 10.13039/501100022664Universidad de Le.

## Funding Statement

No external funding

## Declaration of competing interest

The authors declare that they have no known competing financial interests or personal relationships that could have appeared to influence the work reported in this paper.
