# Supplementary material for: Impact of ChatGPT usage on nursing students education: A cross-sectional study
Source: Heliyon. 2024 Dec 31;11(1):e41559. doi: 10.1016/j.heliyon.2024.e41559 (PMC11755058; doi:10.1016/j.heliyon.2024.e41559)
Supplement: Multimedia component 1 [file mmc1.docx]

# Evaluation Questionnaire on the Use of ChatGPT in Learning Care Management

This questionnaire aims to evaluate nursing undergraduate students' perceptions regarding the usefulness of ChatGPT as a support tool for learning. Your responses will remain confidential and will contribute to improving the course.

We sincerely appreciate your participation.

DEMOGRAPHIC DATA

1. **Age:** _____________________________________
2. **Gender:**

Select one option only:

- Male
- Female
- Other
- Prefer not to say

1. **Employment Status**

Select one option only:

- Full-time student
- Student and part-time worker
- Student and full-time worker
- Other: __________________________________

1. **Place of Residence:**

Select one option only:

- Urban area
- Suburban area
- Rural area

1. **Average Academic Performance (GPA or Grade):**

Select one option only:

- Less than 5
- 5-6
- 6-7
- 7-8
- 8-9
- 9-10

1. **Hours Dedicated to Studying per Week:**

Select one option only:

- Less than 5 hours
- 5-10 hours
- 10-15 hours
- More than 15 hours

EXPERIENCE WITH CHAT GPT

1. **Before this course, had you ever used tools similar to ChatGPT?**

Select one option only:

- Yes (Skip to question 8)
- No (Skip to question 9)

**OPTION 1**

1. **What had you used ChatGPT for previously? (You can select more than one option)**

Select all that apply:

- Support with academic tasks
- Content generation (writing, ideas, etc.)
- General information
- Solving specific problems
- Other: __________________________________________

**OPTION 2**

1. **What other content-generating artificial intelligence tools do you know and have used? (You can select more than one option)**

Select all that apply:

- Image generators (e.g., DALL-E)
- Video generators (e.g., Synthesia)
- Music generators (e.g., Amper Music)
- None
- Other: __________________________

USEFULNESS OF CHATGPT IN THE MANAGEMENT COURSE

1. **Do you consider ChatGPT to have been useful for better understanding management concepts in this course?**

Select one option only:

- Very useful
- Useful
- Neutral
- Not very useful
- Not useful at all

1. **For which aspects of the management course have you used ChatGPT?: (You can select more than one option)**

Select all that apply:

- Understanding theoretical concepts
- Solving practical cases
- Exam preparation
- Completing academic assignments
- Other: ____________________________

1. **Do you consider that using ChatGPT has improved your academic performance in the management course?:**

Select one option only:

- Yes, significantly
- Yes, to some extent
- No, there have been no changes
- No, it has hindered my performance
- I am not sure

1. **Has ChatGPT helped you better relate management concepts to professional nursing practice?**

Select one option only:

- Yes, significantly
- Yes, to some extent
- No, there have been no changes
- No, it has hindered the relationship
- I am not sure

1. **Have you used ChatGPT to understand concepts from other courses besides management?**

Select one option only:

- Yes (Skip to question 15)
- No (Skip to question 16)

1. **If yes, for which other courses have you used ChatGPT?:(You can select more than one option)**

Select all that apply:

- Anatomy
- Physiology
- Pharmacology
- Adult Nursing
- Other: __________________

1. **Do you consider that using ChatGPT has been useful for understanding concepts in these other courses?**

Select one option only:

- Yes, very useful
- Yes, useful
- Neutral
- Not very useful
- Not useful at all
- Use of ChatGPT for Practicum

1. **Do you consider that using ChatGPT will be useful during your nursing practicum at the hospital?**

Select one option only:

- Yes, very useful
- Yes, useful
- Neutral
- Not very useful
- Not useful at all

1. **For which aspects of your nursing practicum do you think ChatGPT could be most useful?: (You can select more than one option)**

Select all that apply:

- Resolving clinical doubts
- Improving communication with patients
- Quick access to medical information
- Support in decision-making
- Clinical documentation
- Other:_________________________

1. **Do you think ChatGPT will be useful for your future professional nursing practice when you start working?:**

Select one option only:

- Yes, very useful
- Yes, useful
- Neutral
- Not very useful
- Not useful at all

1. **For which aspects of your future professional practice do you think ChatGPT will be most beneficial? (You can select more than one option)**

Select all that apply:

- Support in clinical decision-making
- Quick access to updated medical information
- Improving documentation efficiency
- Assistance in patient education and communication
- Training and continuous education
- Other:_____________________

1. **Have you used ChatGPT to improve specific skills needed for your future professional career?**

Select one option only:

- Yes (Skip to question 22)
- No (Skip to question 23)

1. **If yes, which specific skills have you improved using ChatGPT?: (You can select more than one option)**

Select all that apply:

- Patient communication
- Time management
- Clinical documentation
- Clinical decision-making
- Other:__________________________

1. **Do you consider ChatGPT to be a useful tool for continuing education in nursing?:**

Select one option only

- Yes
- No
- I am not sure

1. **Have you recommended or would you recommend the use of ChatGPT to colleagues or professionals in the nursing field?:**

Select one option only

- Yes, I have already recommended it
- Yes, I would recommend it
- No, I would not recommend it

**GENERAL SATISFACTION**

1. **On a scale of 1 to 5, how would you rate your overall satisfaction with the use of ChatGPT in this course?:**

Select one option only:

- 1 (Very dissatisfied)
- 2 (Dissatisfied)
- 3 (Neutral)
- 4 (Satisfied)
- 5 (Very satisfied)

1. **Would you recommend the use of ChatGPT to other nursing students?:**

Select one option only

- Yes
- No

1. **Did you enjoy using ChatGPT in the management course?:**

Select one option only

- Yes, I liked it a lot
- Yes, I liked it
- Neutral
- No, I didn’t like it
- No, I didn’t like it at all

1. **Do you think the use of ChatGPT has made the management course more enjoyable?:**

Select one option only

- Yes, much more enjoyable
- Yes, more enjoyable
- Neutral
- No, it hasn’t made a difference
- No, it has made it less enjoyable

1. **Do you think the use of ChatGPT has helped you better face the challenges of the management course?:**

Select one option only

- Yes, significantly
- Yes, to some extent
- No, there have been no changes
- No, it has made the challenges more difficult
- I am not sure

1. **Do you consider that the use of ChatGPT has facilitated your understanding of study materials compared to traditional methods?:**

Select one option only

- Yes, significantly
- Yes, to some extent
- Neutral
- No, there hasn’t been a difference
- No, it has been more difficult

1. **Has the use of ChatGPT helped improve your motivation to study the management course?:**

Select one option only:

- Yes, significantly
- Yes, to some extent
- No, there have been no changes
- No, it has decreased my motivation
- I am not sure

**KNOWLEDGE AND USE AT HOME**

1. **Do your family members (siblings, parents, etc.) know about artificial intelligence tools like ChatGPT?:**

Select one option only

- Yes
- No
- I am not sure

1. **Have your family members ever used these tools?:**

Select one option only:

- Yes
- No
- I am not sure

1. **Have you talked to your family members about these tools?:**

Select one option only

- Yes
- No

1. **Have any of your family members shown interest in learning more about ChatGPT or other artificial intelligence tools?:**

Select one option only:

- Yes, a lot of interest
- Yes, some interest
- Neutral
- No, little interest
- No, no interest

1. **Have you taught any of your family members to use ChatGPT or other artificial intelligence tools?:**

Select one option only

- Yes
- No

1. Do your family members use ChatGPT or other artificial intelligence tools for their work or studies?

Select one option only

- Yes
- No
- I am not sure
- Please provide any additional comments about your experience using ChatGPT and suggestions for improving its use in the future:

We sincerely appreciate your participation in this survey. Your responses will be highly valuable for improving the course in future editions and for assessing the use of AI tools in more contexts within the course.
